# Supplementary figures and images for: Cell-surface protein YwfG of Lactococcus lactis binds to α-1,2-linked mannose
Source: PLoS One. 2023 Jan 5;18(1):e0273955. doi: 10.1371/journal.pone.0273955 (PMC9815576; doi:10.1371/journal.pone.0273955)

A

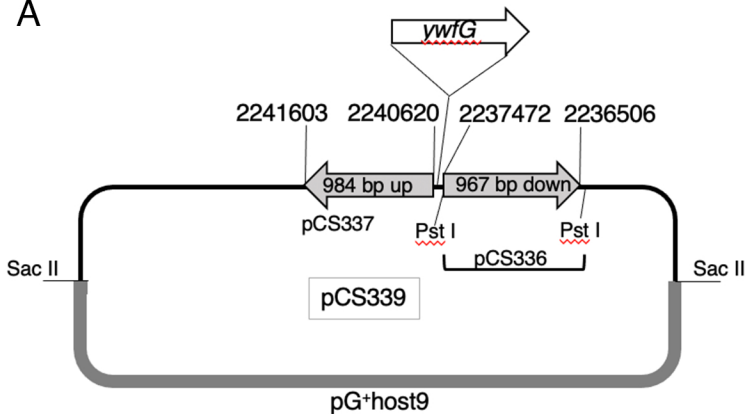

B

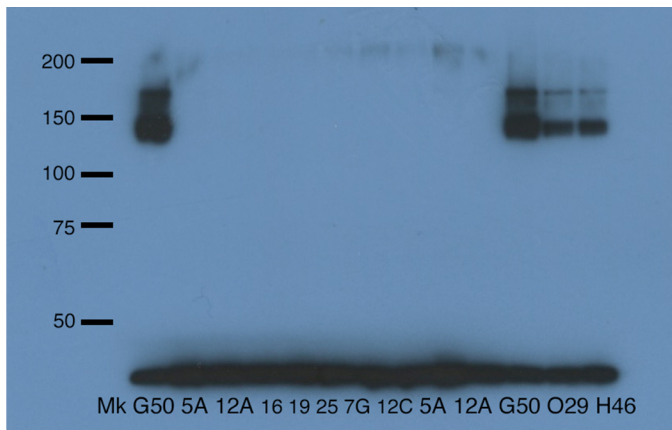

Supplement: S1 Fig — A, Map of the pCS339 plasmid for ywfG deletion. To construct this plasmid, a 984-bp upstream fragment (2240620–2241603) and a 991-bp downstream fragment (2236506–2237496) of the ywfG gene (LLG50_11005, complementary 2237254–2240577) were amplified by PCR; each fragment was ligated into the pGEM-T Easy plasmid, resulting in pCS336 and pCS337, respectively. The PstI fragment (967 bp) of pCS336 was ligated into the PstI site of pCS337, resulting in pCS338. SacII–digested pCS338 and pG+host9 were ligated, resulting in pCS339. B, Western blots of the cell-wall proteins of L. lactis G50, O29, H46, and ΔywfG strains. (PDF) [file pone.0273955.s001.pdf]

A

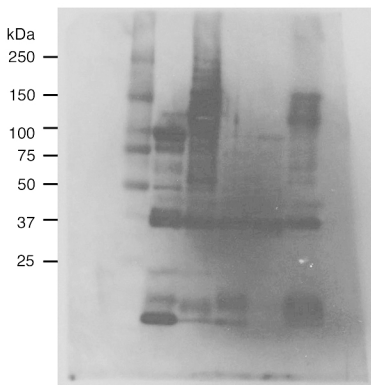

MK S63 G50 P79 342 O29

B

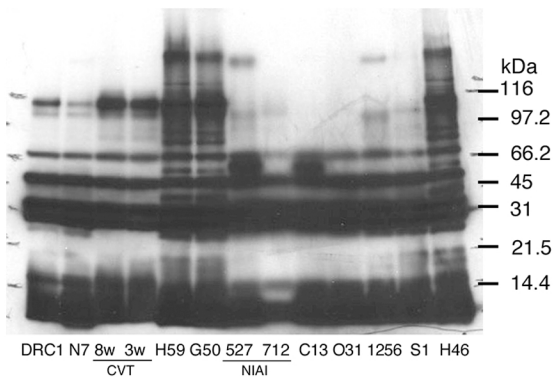

DRC1 N7 8w 3w H59 G50 527 712 C13 O31 1256 S1 H46  
CVT NIAI

Supplement: S2 Fig — A, Western blots of surface-exposed proteins of lactococcal strains S63, G50, P79, 342, and O29. B, Western blots of cell lysates of 13 lactococcal strains. (PDF) [file pone.0273955.s002.pdf]

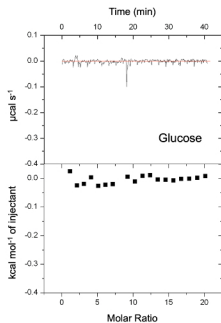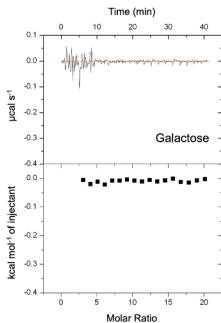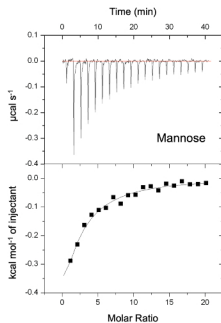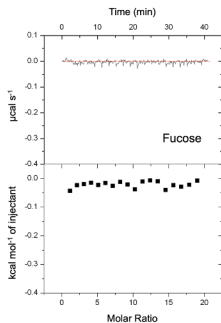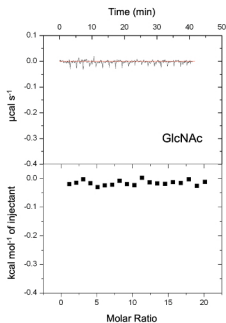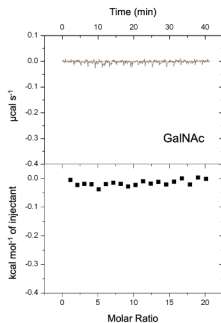

Supplement: S4 Fig — Isothermal titration of YwfG28–270 against D-glucose, D-galactose, D-mannose, D-fucose, GlcNAc, GalNAc. (PDF) [file pone.0273955.s004.pdf]

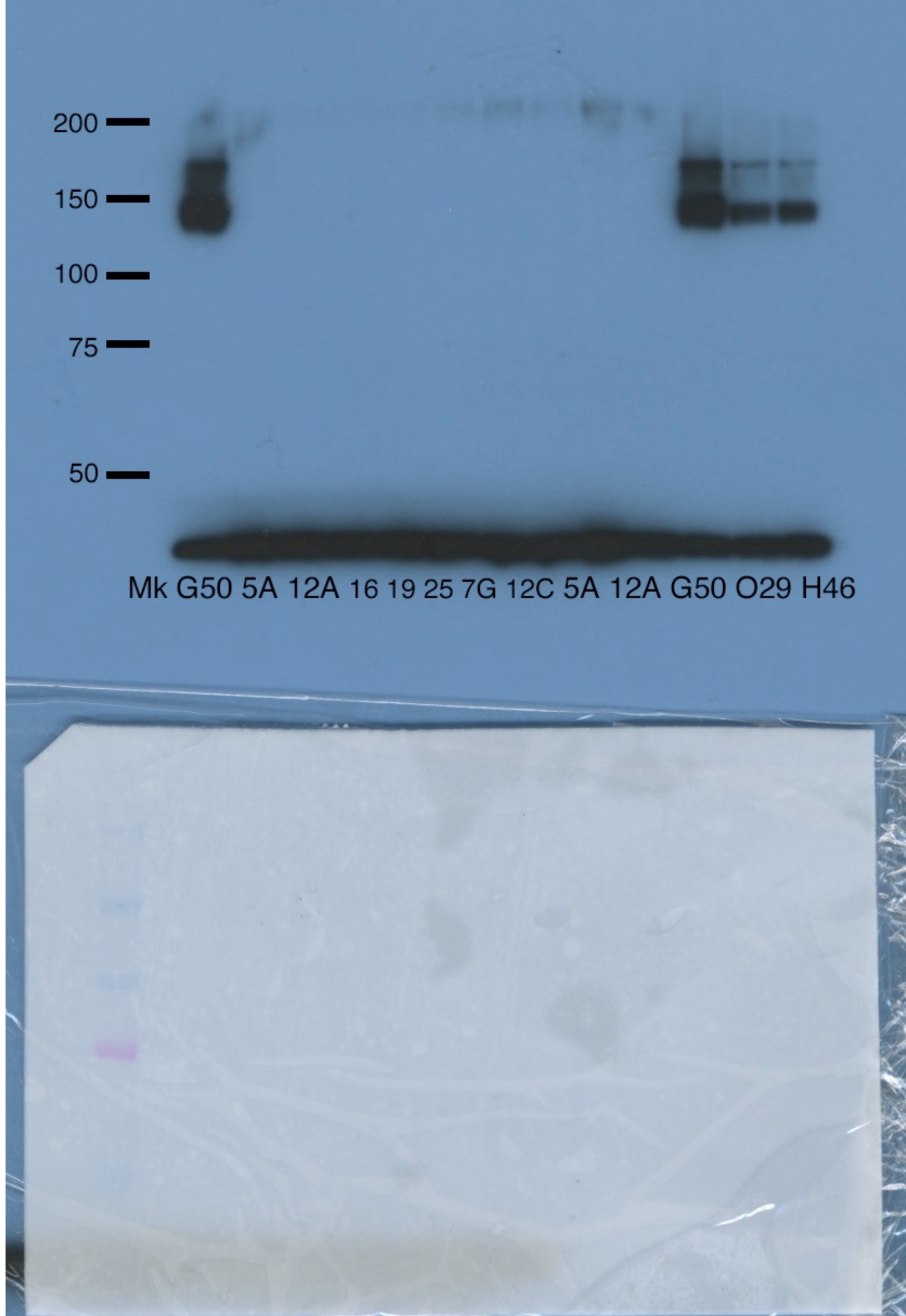

S1\_raw\_Fig image S1B

A

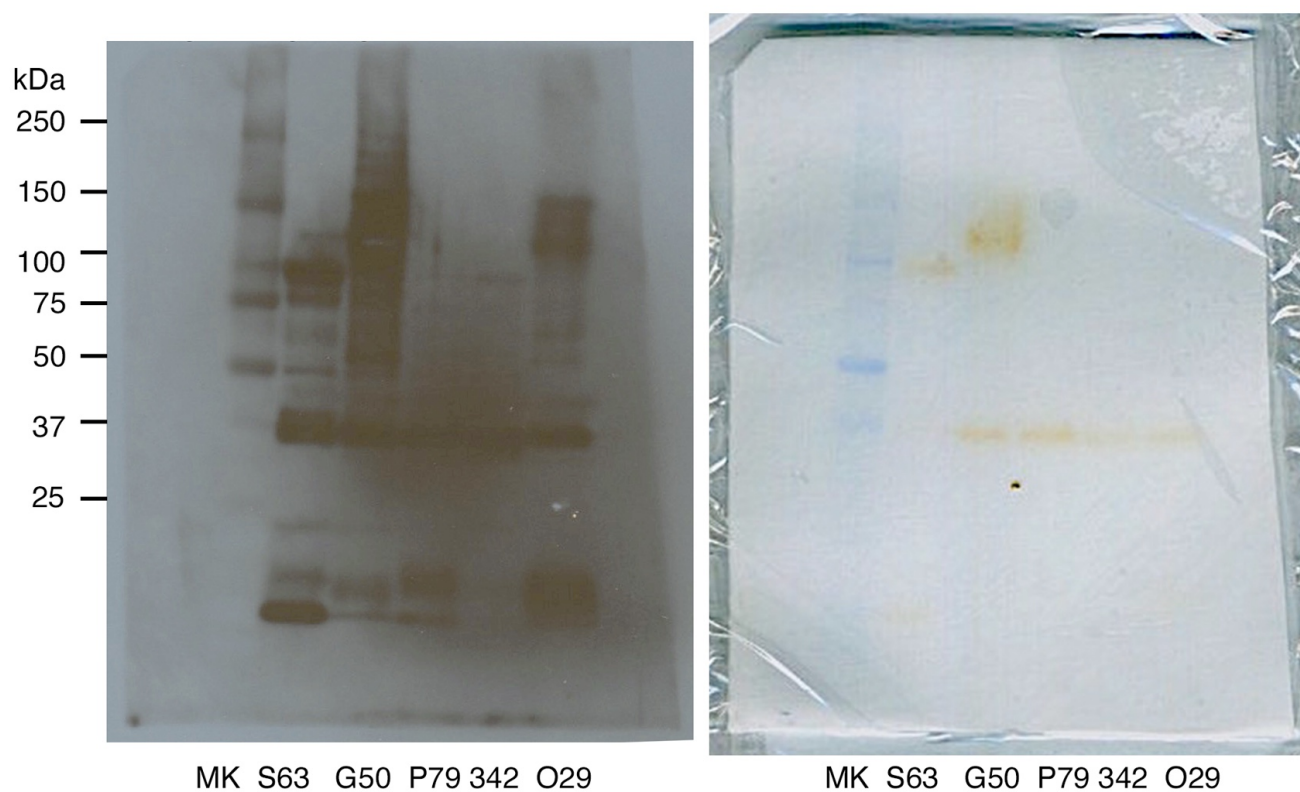

B

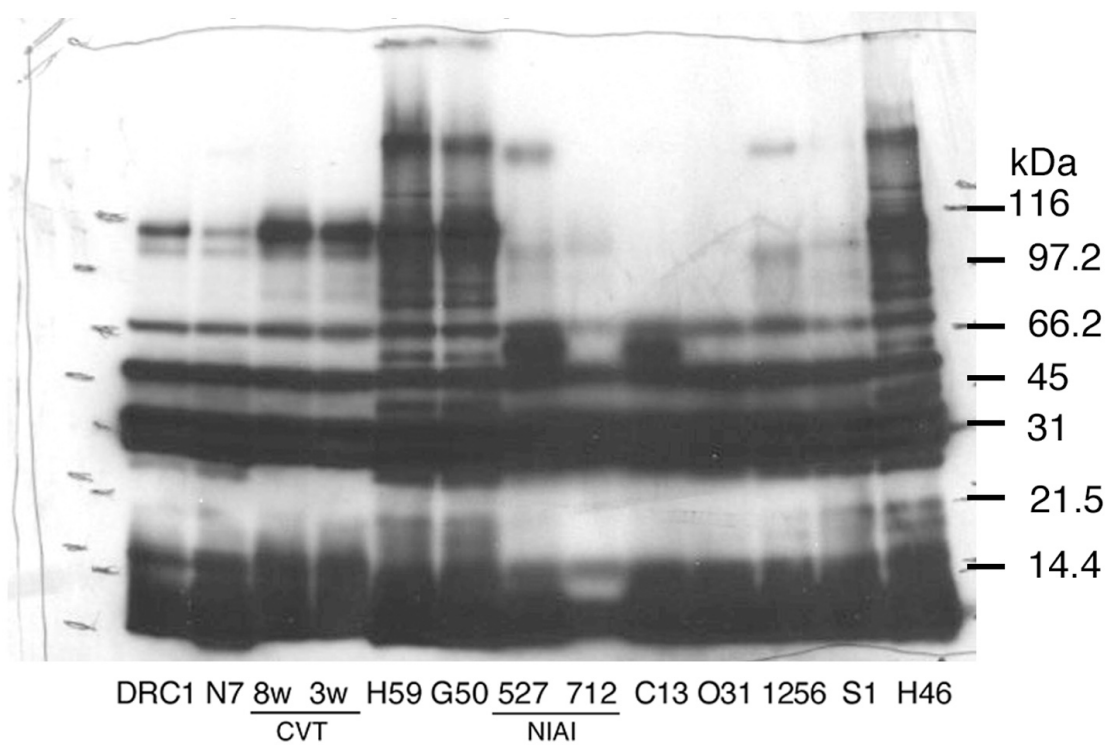

S1\_raw\_Fig images\_S2

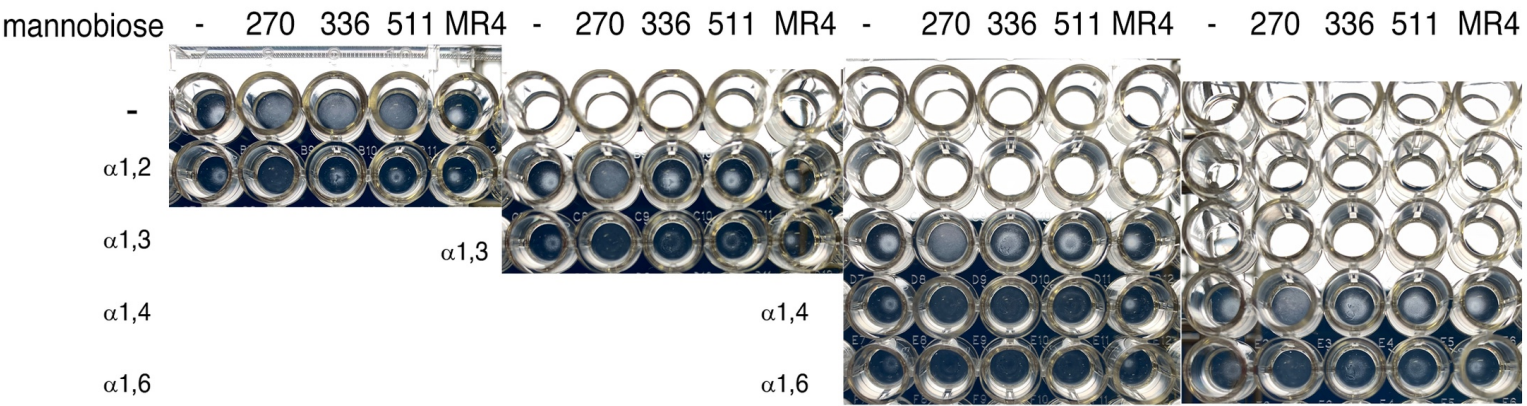

S1\_raw\_Fig image Fig 7C

Supplement: S1 Raw images — S1_raw_Fig image Fig 1: Two gels loaded with the same samples, one for CBB staining and one for western blotting, were simultaneously subjected to SDS-PAGE. Proteins were visualized by CBB staining (left panel, Fig 1B) or western blotting visualizing by exposure with ECL regent (right panel, Fig 1A). The middle panel is the PVDF membrane after exposure with ECL, visualizing the protein on which the ECL reagent bound. S1_raw_Fig image S1B: Western blotting of cell-wall proteins of delta ywfG strains, G50, O29 and H26 visualized by exposure with ECL regent (upper panel, S1B) and the PVDF membrane after exposure with ECL (lower panel). S1_raw_Fig image S2: A Western blotting of cell-surface proteins of S63, G50, P79, 342 and O29 visualized by exposure with ECL regent (left panel, S2A) and the PVDF membrane after exposure with ECL (right panel). B Western blotting of cell lysates of Lactococcal strains visualized by exposure with ECL regent. S2_raw_Fig image Fig 7C. Interaction of yeast cells and YwfG derivatives was observed in a microtiter plate. Photographing yeast cells accumulated at the bottom of the wells requires oblique lighting, and it is difficult to take a photograph all wells at once, so 4 photographs were combined to create Fig 7C. (PDF) [file pone.0273955.s006.pdf]
