# Supplementary material for: Cell-surface protein YwfG of Lactococcus lactis binds to α-1,2-linked mannose
Source: PLoS One. 2023 Jan 5;18(1):e0273955. doi: 10.1371/journal.pone.0273955 (PMC9815576; doi:10.1371/journal.pone.0273955)
Supplement: S3 Fig — Peptides obtained were aligned to the deduced sequence and are shown underlined and in bold. Peptides that were detected on both sides of the trypsin cleavage site are indicated by a triangle. (PDF) [file pone.0273955.s003.pdf]

MNKKSSALLTM**GTLTLLAGGGVVLTNLPDSFKIQR**VYAATSRDITVYPK**DFLTYFQRNGS** 60

AAGFDYDLATYTQTLTPNKASQAGNVTLKTKVDMSQNFTFTGKINLGDKAQNAGGADGVG 120

FLFHPGDTNVVGAPGGAAGIGGVNGAFGFKLDTYYNGVGENSFTPDPSNFK**GKPFGAFVD** 180

GLNGQAKTIASSAQSISEPSNNNFVDFTMSYNGATKVMSVTYGGQTWTQDVSSFVGTNQA 240

MSFSIAASTGAFMNLQQLRNVNFTYTVAQGTVIANYVDEQGNTIAQQETTSGDIDTPYVT 300

SQK**TIPGYTFKASNGAATSGNYAANDQTVNYVYTRNQGSIDVTYIDQTTGQTLSKK**DLSG 360

GTGDSSNYTTADTIKSYTDAGYELVSDNYPSGGTVFTDTAQHYVVNLK**QKLVVSSEQKQV** 420

NETIQYVVYEDGSKAADDYNAPPLNFTRSVTTNQVTGEKTYGDWQAQNGDSFGEVVSPTIK 480

GETADQLK**IDAISGITANSADIQK**KVVYKR**NQGTIDVTYIDETTGQVLTKKDLSGGTDDP** 540

SNYTTADDIKSYTAK**GYELVSDDYPSGGTVFTDEPQHYVVK**LK**HGLTESTDKKAVNQVIH** 600

YVYEGGGEAATDHNATVDFSRTITTDRVTNDKTYGDWTADNGDSFASVTSPVIDGYTADQ 660

LK**VSEMTGITADTEDISVTVTYTR**NQGTIDITYIDQTTGQTLSEKDLSGGTGDDSGYTTA 720

DTIK**SYTDKGYELVSNDYPEDGTKFADDPQHYIVRLKHGLTEVTENK**TVNQVIHYVYEGG 780

GEAATDHNATVVFSQTITTDKVTGEKTYSDWTADNGDSFASVTSPVIDGYTADQLKVSEM 840

TGITVDTEDISVTVTYTR**NQGTIDVTYIDETTGK**ILTTK**DLSGGTGDDSGYTTADTIKSY** 900

TDKGYELVSNDYPEDGTKFADDPQHYIVRLKHGTVVETENKSVNEVIHYVYDNGDKAADN 960

YKATIVFSRTITTDKVTGEKTYSDWTADNGGRFAAVLSPIIKDYIASQLK**IDEMTGITVD** 1020

TADIERIVVYHKVPAGIIVPPVHPDKPSQPSNNQSKTPTAKAVK**DSKPTDVLPSTGDSQK** 1080

SQIVLTLLGIMAVIISPLALLLRRRKQ 1107
